# MicroRNA-21 mediated cross-talk between cardiomyocytes and fibroblasts in patients with atrial fibrillation

**DOI:** 10.3389/fcvm.2023.1056134

**Published:** 2023-02-17

**Authors:** Kabita Pradhan, Paul Niehues, Balram Neupane, Carole Maleck, Ahmad Sharif-Yakan, Mahdi Emrani, Matthias Daniel Zink, Andreas Napp, Nikolaus Marx, Michael Gramlich

**Affiliations:** ^1^Department of Cardiology, University Hospital RWTH Aachen University, Aachen, Germany; ^2^Department of Cardiology and Cardiovascular Diseases, Eberhard Karls University, Tübingen, Germany

**Keywords:** miR-21, atrial fibrillation, fibrosis, low-voltage area, mocetinostat

## Abstract

**Background:**

Atrial fibrosis represents a major hallmark in disease progression of atrial fibrillation (AF). We have previously shown that circulating microRNA-21 (miR-21) correlates with the extent of left atrial fibrosis in patients undergoing catheter ablation for AF and can serve as a biomarker to predict ablation success. In this study, we aimed to validate the role of miR-21-5p as a biomarker in a large cohort of AF patients and to investigate its pathophysiological role in atrial remodeling.

**Methods:**

For the validation cohort, we included 175 patients undergoing catheter ablation for AF. Bipolar voltage maps were obtained, circulating miR-21-5p was measured, and patients were followed-up for 12  months including ECG holter monitoring. AF was simulated by tachyarrhythmic pacing of cultured cardiomyocytes, the culture medium was transferred to fibroblast, and fibrosis pathways were analysed.

**Results:**

73.3% of patients with no/minor LVAs, 51.4% of patients with moderate LVAs and only 18.2% of patients with extensive LVAs were in stable sinus rhythm (SR) 12  months after ablation (*p* < 0.01). Circulating miR-21-5p levels significantly correlated with the extent of LVAs and event-free survival. *In-vitro* tachyarrhythmic pacing of HL-1 cardiomyocytes resulted in an increased miR-21-5p expression. Transfer of the culture medium to fibroblasts induced fibrosis pathways and collagen production. The HDAC1 inhibitor mocetinostat was found to inhibit atrial fibrosis development.

**Conclusion:**

We validated miR-21-5p as a biomarker that reflects the extent of left atrial fibrosis in AF patients. Furthermore, we found that miR-21-5p is released *in-vitro* from cardiomyocytes under tachyarrhythmic conditions and stimulates fibroblasts in a paracrine mode to induce collagen production.

## 1. Introduction

Atrial fibrillation (AF) is the most common cardiac arrhythmia with a life-time risk of 1 in 3 individuals in Europe (1). AF accounts for 20–30% of all strokes and can also lead to congestive heart failure, cognitive dysfunction, cardiovascular morbidity and mortality. Its prevalence is more than 9% for those above 80 years compared to 0.1% for those under 55 years (2, 3). A common theory states that ectopic foci located in the pulmonary veins trigger the arrhythmia, whereas its perpetuation is mediated by atrial fibrosis in the atria themselves, serving as the substrate that creates multiple functional and structural re-entry mechanisms (4, 5). Importantly, frequent AF episodes fuel the development of adverse atrial remodeling with substrate creation (“AF begets AF”). It is well established that the extent of left atrial fibrosis is a key negative predictor for AF recurrence after catheter ablation (6, 7).

MicroRNAs, non-coding, small (18–22 nucleotides long), and conserved RNAs have been known to play crucial roles in the pathogenesis of cardiac fibrosis. Among numerous microRNAs, microRNA-21 (miR-21) plays an exceptional role in the pathogenesis of tissue fibrosis. miR-21 signaling is critical in atrial fibrotic remodeling of an AF rat model (8), in lung fibrosis (9) and after myocardial ischemia in mice (10).

We have previously shown that circulating miR-21 is upregulated in patients with AF and that its serum concentration measured in blood samples obtained from the left atrium (LA) correlates with the extent of left atrial low voltage areas (LVAs) (11), which give an estimation of fibrosis (12). MiR-21 strongly correlate to treatment outcome after catheter ablation of AF, thereby serving as a biomarker to predict potential treatment success.

In this study, we aimed to validate the role of miR-21-5p (here onwards referred as miR-21) as a biomarker for AF in a large cohort of patients by assessing miR-21 concentrations and correlating them to left atrial fibrosis. In addition, we analyzed the pathophysiological role of miR-21 *in-vitro* using disease-modeling with cultured cardiomyocytes and fibroblasts.

## 2. Materials and methods

### 2.1. Patients

A total of 175 consecutive patients >18 years old who presented for catheter ablation due to AF (paroxysmal and persistent) at the university hospital RWTH Aachen were included in this study. 165 patients had their first ablation for atrial fibrillation, 10 had a re-do ablation. All patients gave their informed consent. The study was approved by the local ethics committee (EK054/21). All clinical characteristics of patients are listed in Table 1.

**Table 1:** Demographic and clinical characteristics of AF patients included in the study.

Age, year	66.9 ± 8.9
Male	109 (62.2%)
BMI, kg/m^2^	28.8 ± 7.3
Fibrosis stage (left atrial LVA)	
0–10%	65 (37.14%)
10–30%	39 (22.29%)
>30%	71 (40.57%)
EHRA-Score	2.3 ± 0.65
CHA_2_DS_2_-VASc score	2.7 ± 1.5
Left atrial diameter, mm	42 ± 2.1
Systolic blood pressure, mmHg	127 ± 12.4
LV ejection fraction, %	51.3 ± 8.2
Mitral valve disease (>first degree)	15 (8.5%)
Previous stroke	16 (9.1%)
Previous PCI	31 (17.7%)
Previous myocardial infarction	17 (9.7%)
Previous CABG	5 (2.9%)
LV hypertrophy	49 (28%)
Chronic kidney disease	44 (25.1%)
Type 2 diabetes mellitus	32 (18.2%)
OSAS	16 (9.1%)
Hyperlipidemia	100 (57.1%)
Ablation strategy	
PVI RF	175 (100%)
Left atrial roof line	41 (23.4%)
Mitral line	60 (34.2%)
CFAE ablation	1 (0.57%)
RA isthmus line	93 (53.1%)

a AF, Atrial fibrillation; BMI, Body mass index; LVA, Low-voltage area; EHRA, European heart rhythm association; CHA2DS2-VASc, Congestive heart failure, hypertension, age ≥ 75, diabetes mellitus, stroke (double point), vascular disease, age 65–74 years (double point) and sex category (female); LV, Left ventricle; PCI, Percutaneous coronary intervention; CABG, Coronary artery bypass grafting; OSAS, Obstructive sleep apnoea syndrome; PVI, Pulmonary vein isolation; RF, Radiofrequency; CFAE, Complex fractionated electrogram; RA, Right atrium.

### 2.2. Mapping

**Figure 1:**
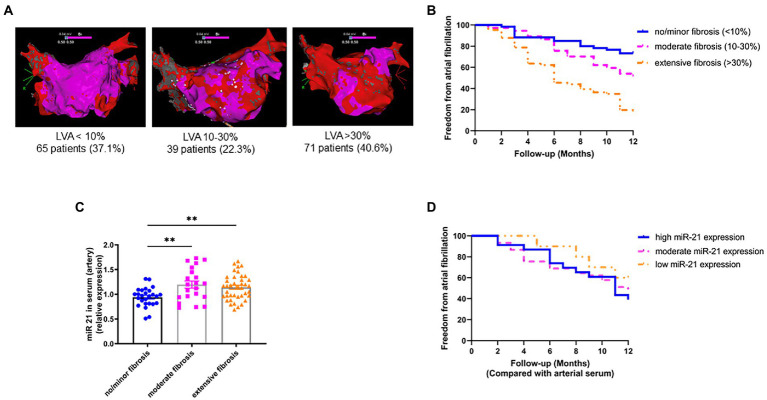
Circulating miR-21 correlates with left atrial LVAs and treatment outcome. **(A)** Representative high-density bipolar voltage maps from patients undergoing AF catheter ablation. No/minor LVAs: 37.1%, moderate LVAs: 22.3%, Extensive LVAs: 40.6% (semi-quantitative illustration, no scale bar available). **(B)** Left atrial LVAs strongly correlate with treatment outcome after ablation. Patients in SR after 12  months: 73.3% having no/minor LVAs, 51.4% having moderate LVAs and 18.2% having extensive LVAs. *n* = 37–66. **(C)** Circulating miR-21 positively correlates with left atrial LVAs. Patients with low miR-21 serum concentration had low LVAs and patients with high miR-21 had significantly more LVAs. *n* = 21–40. **(D)** Circulating miR-21 in arterial serum correlates with treatment outcome after catheter ablation. 39.1% of patients with high miR-21 serum levels were in stable SR 12  months after ablation, whereas 48.9% with moderate miR-21 concentration and 60% with low miR-21 concentration were free from AF 12 months after ablation. *n* = 10–45. Data are expressed as mean ± SEM. **p < 0.01. LVAs = low voltage area.

After transseptal puncture, an electrical cardioversion was performed to achieve sinus rhythm (SR). A high-density 3D- anatomical voltage map in SR was obtained, using the Carto3 System (Johnson & Johnson) with a Pentaray high density mapping catheter. A minimum of 6,000 voltage points were obtained per patient. A local electrogram of <0.5 mV during SR was attributed as LVA. The Carto-3 built-in software was used to remove the pulmonary veins and the mitral anulus. The percentage of LVAs was then calculated from the surface of the left atrial corpus. In case of a re-do ablation, lesions generated from the first PVI were excluded for calculation of LVAs. Maps shown in Figure 1A are color-coded in red (<0.5 mV, substantial LVAs) or purple (>0.5 mV, normal voltage). Patients were divided into three groups according to the extents of LVAs: group A (<10% LVAs), group B (10–30% LVAs), and group C (>30% LVAs). Prior to ablation, 20 ml blood was drawn from a peripheral artery (radial artery) and a peripheral vein (femoral vein) and processed for microRNA isolation. The miR-21 concentration was determined as described previously (11).

### 2.3. Ablation

All patients considered for ablation underwent pulmonary vein isolation with a force-controlled irrigated radiofrequency ablation catheter (Thermocool Smarttouch SF, Johnson & Johnson). Additional ablation strategies targeting the substrate were allowed if indicated by the operator. Detailed follow up procedures is available in the Supplementary material.

### 2.4. *In-vitro* AF model

The medium of confluent cultured murine atrial cardiomyocytes (HL-1 cells) was substituted with supplemented Claycomb medium with reduced (2%) FBS and paced with 1 Hz (simulating SR) or 5 Hz, 25 V, 6 ms with an 80% variability (simulating AF) for 4.5 h in a 6 well C-dish culture dish using the c-Pace EP cell culture stimulator (Ionoptix). The voltage was confirmed by Rohde and Schwarz RTO 1024 Oscilloscope/2Ghz 10 GS a/s. A control group was maintained under unstimulated conditions. After 4.5 h, the medium was centrifuged at 1,200 rpm for 5 min and the supernatant was transferred to NIH/3T3 fibroblasts. Pathway analysis was performed using a profiler PCR array (RT2 profiler PCR Array mouse fibrosis 96 well format, Qiagen), western blot and immunofluorescence. A luciferase reporter assay under the control of a miR-21 promoter was used for drug screening with selected FDA-approved drugs. Expanded methods section is available in the Supplementary material.

### 2.5. Statistical analysis

For patient sample analysis, univariable cox regression analysis was performed to correlate recurrence of arrhythmia to individual clinical covariates. Multivariable linear regression analyses were performed to correlate miR-21 expression and left atrial LVA for recurrence of arrhythmia after adjusting for the covariates: sex, age, congestive heart failure, diabetes mellitus, mitral valve disease and hypertension. All risks were estimated using the Kaplan–Meier estimator with 95% confidence intervals. All other values for *in-vitro* experiments are expressed as mean ± SEM. Student’s *t*-test or one-way ANOVA were performed. Data were analysed using GraphPad Prism.

## 3. Results

### 3.1. LVAs and miR-21 expression correlates with ablation success in AF patients

High density 3D-anatomical voltage mapping of patients undergoing catheter ablation for AF (all-comer collective at the university hospital RWTH Aachen, Germany) showed that 37.1% had no or only minor LVAs (group A, <10% of the LA surface), 22.3% had moderate LVAs (group B, 10–30% of the LA surface) and 40.6% had extensive LVAs (group 3, >30% of the LA surface; Figure 1A). Successful pulmonary vein isolation was achieved in all patients. An additional left atrial roof line ablation was performed in 23.4%, a mitral line in 34.2%, and a right atrial isthmus line in 53.1% of patients. Ablation of complex fractionated electrograms was performed in 0.6% of cases. All baseline characteristics and the procedure details are listed in Table 1.

We found a strong correlation between LVAs and outcome after catheter ablation. 73.3% of patient with no/minor LVAs, 51.4% of patients with moderate LVAs and only 18.2% of patient with extensive LVAs were in stable SR 12 months after ablation (Figure 1B). After adjustment for multiple confounders, a 1% increase in LVA resulted in a 1.021 (1.013–1.029. *p* < 0.01) hazard ratio of AF recurrence (Table 2).

**Table 2:** Clinical characteristics and risk factor associated with AF patients.

	Hazard ratio	95% confidence interval	*p*-value
AF recurrence per 1 AU increase in miR-21 (artery)	1.104	0.398–3.063	0.850
AF recurrence per 1 AU increase in miR-21 (vein)	0.191	0.083–0.440	<0.01*
AF recurrence per 1% increase in LVAs	1.021	1.013–1.029	<0.01*
Male sex	0.94	0.573–1.543	0.807
Age (per year)	1.005	0.979–1.031	0.718
BMI (per unit)	1.016	0.970–1.064	0.502
Previous PCI/CABG	1.118	0.610–2.050	0.717
Previous myocardial infarction	1.052	0.455–2.434	0.905
LV hypertrophy	1.106	0.655–1.866	0.707
Chronic kidney disease	1.607	0.970–2.663	0.066
Hypertension	1.474	0.878–2.475	0.142
CHA2DS2-VASc score (per point)	1.032	0.877–1.216	0.702
LVEF, %	0.969	0.941–0.997	0.028*
LA size, mm	1.075	1.021–1.133	<0.01*
OSAS	0.817	0.328–2.034	0.664
Mitral valve disease	1.997	0.985–4.051	0.055
Diabetes mellitus	0.973	0.510–1.858	0.934

b AF, Atrial fibrillation; AU, Arbitrary unit; miR-21, microRNA-21; LVA, Low-voltage area; PCI, Percutaneous coronary intervention; CABG, Coronary artery bypass grafting; LV, Left ventricle; EHRA, European heart rhythm association; LVEF, Left ventricular ejection fraction; LA, Left atrium; OSAS, Obstructive sleep apnoea syndrome. *p < 0.05.

Patients were assigned in 3 groups according to their miR-21 serum concentration: group I with very high relative miR-21 expression (>1.20 AU), group II with intermediate relative miR-21 expression (0.80–1.20 AU), and group III with very low miR-21 serum concentration (<0.80 AU). We found a significant positive correlation between LVAs and circulating miR-21 (in arterial serum; Figure 1C). After adjusting multiple confounders, an increase of 1 AU miR-21 (arterial serum) concentration resulted in a 1.104 (0.398–3.063, *p* < 0.850) hazard ratio of AF recurrence (Table 2).

Since presence of left atrial LVAs is strongly correlated with treatment outcome and LVAs are correlated with miR-21 serum concentrations, we also found a correlation between miR-21 and treatment outcome. Figure 1D shows the Kaplan–Meier curve on event-free survival after ablation of AF. 39.1% of patients in group I were in stable SR during the observation period, whereas 48.9% of patients in group II and 60.0% patients in group III had SR 12 months after ablation.

### 3.2. Irregular tachyarrhythmic pacing of HL1 cardiomyocytes leads to increased miR-21 expression

**Figure 2:**
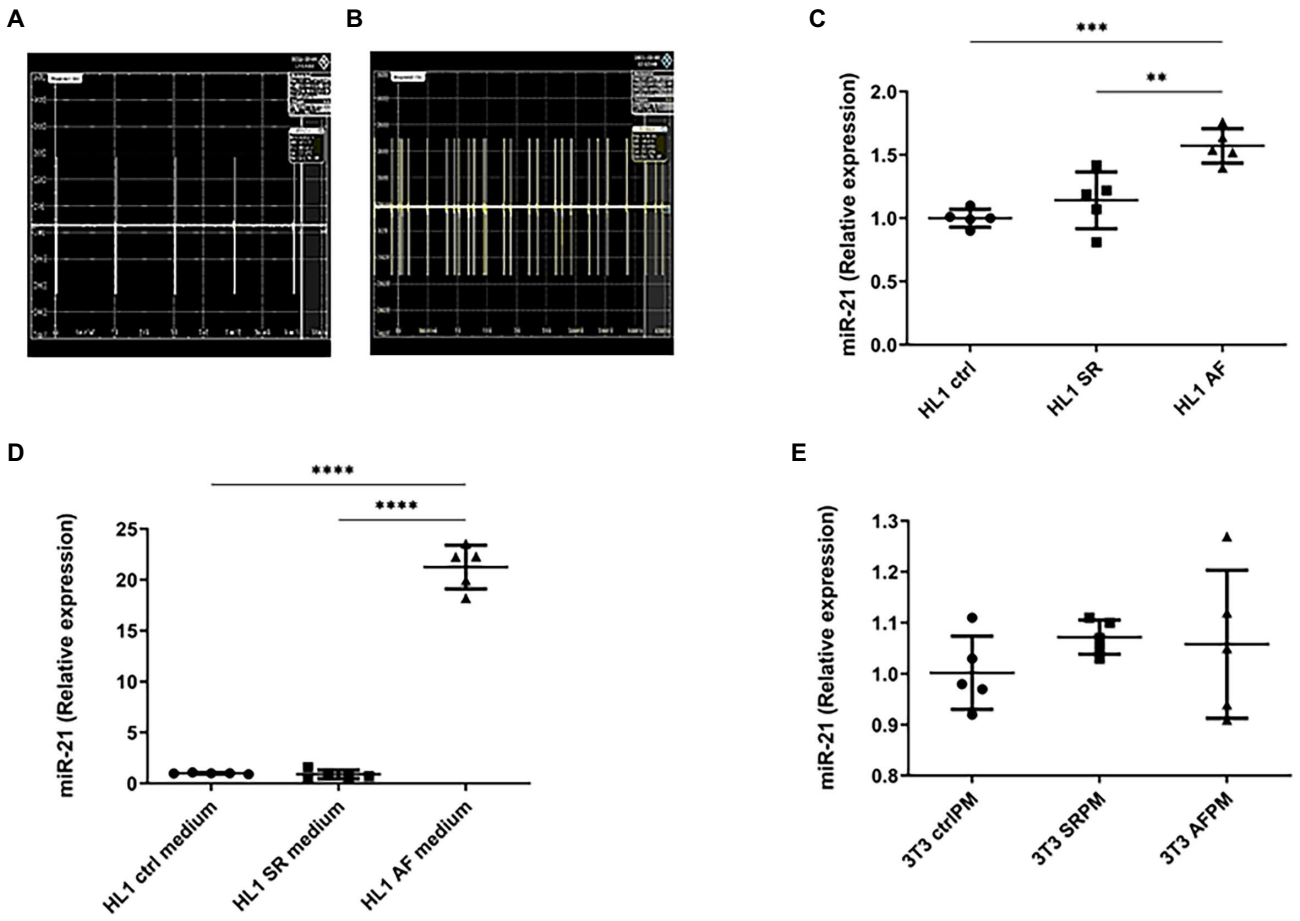
AF model with cultured murine cardiomyocytes (HL-1 cells) and fibroblast (NIH/3 T3 cells). **(A)** Sinus rhythm was simulated using a regular wave form at 25 V 6 ms 1 Hz. **(B)** For the simulation of AF, a faster, irregular pacing protocol was used (25 V 6 ms 5 Hz, 80% variability). **(C)** After 4.5 h of pacing, increased miR-21 expression under tachyarrhythmic conditions could be observed in the HL-1 cardiomyocytes. **(D)** miR-21 concentration in the medium of cardiomyocytes that were held under tachyarrhythmic conditions was strongly increased, whereas no release of miR-21 to the medium under SR conditions could be observed. **(E)** No increase in miR-21 expression in NIH/3 T3 fibroblast was observed, when treated with control, SR and AF paced medium from HL1 cardiomyocyte cells. **(C–E)** Representative data out of 3 similar experiments. Data are expressed as mean ± SEM. ****p < 0.0001, ***p < 0.001, **p < 0.01. CtrlPM, control pacing medium; SRPM, sinus rhythm pacing medium; AFPM, atrial fibrillation pacing medium.

To study the underlying mechanisms of miR-21 mediated fibrosis, murine atrial cardiomyocytes (HL1 cells) were either paced regularly (simulating SR; Figure 2A) or with irregular elevated frequency simulating AF (Figure 2B). After 4.5 h of pacing, miR-21 was found to be overexpressed only in the cardiomyocytes of AF paced group (Figure 2C) and released to the medium (Figure 2D). A 21-fold increment of miR-21 expression was observed in AF paced cells medium compared to SR (*p* < 0.0001). The medium from paced (AF and SR) or control HL1 cardiomyocytes was transferred to NIH/3T3 fibroblast and incubated. Relative expression of miR-21 in the fibroblasts, 24 or 72 h after the medium transfer, was relatively constant (Figure 2E).

### 3.3. miR-21 stimulates fibroblasts while anti-miR-21 prevents the activation of fibroblasts for collagen production

**Figure 3:**
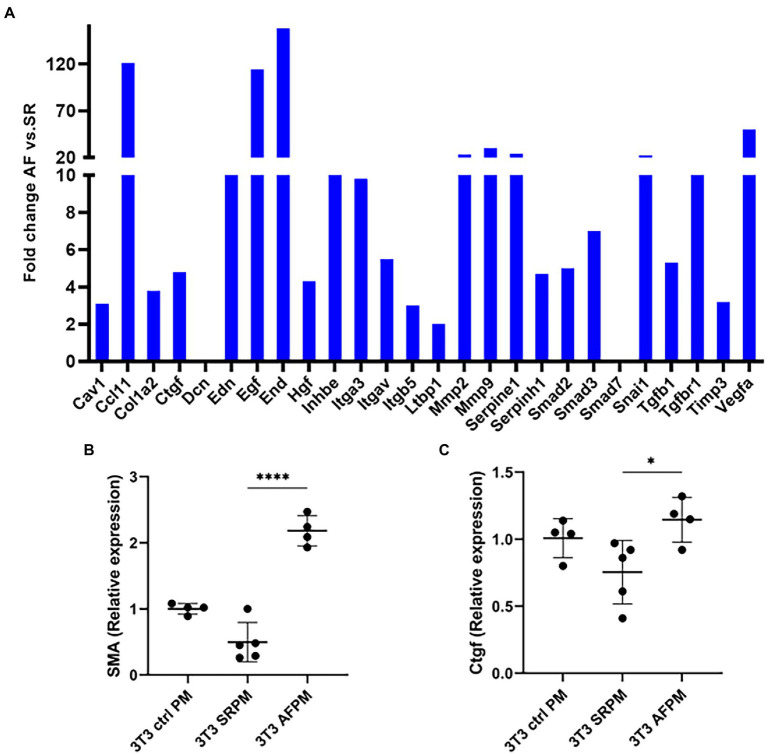
miR-21 released from cardiomyocytes induces pro-fibrotic pathways in NIH/3 T3 fibroblasts. **(A)** The medium of tachyarrhythmic paced cardiomyocytes was transferred to fibroblasts and incubation was done for 72 h, then the pathway analysis was performed. A fibrosis profiler array showed a strong induction of pro-fibrotic pathways and collagen expression in the NIH/3 T3 cells incubated with AF medium. Each sample type represents a pool of one 6 well plate. The induction is especially true for the fibrosis markers α-SMA **(B)** and Ctgf **(C)**. **(B,C)** Representative data out of 3 similar experiments. Data are expressed as mean ± SEM. ****p < 0.0001, *p < 0.05. Cav1: caveolin-1, Ccl11: c-c motif chemokine 11, Col1a2: collagen type I alpha 2, Ctgf: connective tissue growth factor, Dcn: decorin, Edn: endothelin, Egf: epidermal growth factor, End: endoglin, Hgf: hepatocyte growth factor, Inhbe: inhibin subunit beta E, Itga3: integrin subunit alpha3, Itgav: integrin alpha V, Itgb5: integrin beta 5, Ltbp1: latent transforming growth factor-β-binding protein-1, Mmp2: matrix metalloproteinase-2, Mmp9: matrix metalloproteinase-9, Serpine1: serpin family E member 1, Serpinh1: serpin family H member 1, Smad2: SMAD family member 2, Smad3: SMAD family member 3, Smad7: SMAD family member 7, Snai1: snail family transcriptional repressor 1, Tgfb1: transforming growth factor beta 1, Tgfbr1: transforming growth factor beta receptor 1, Timp3: tissue inhibitor of metalloproteinases-3, Vegfa: vascular endothelial growth factor a, α-SMA: alpha smooth muscle actin, Ctrl PM: control pacing medium, SRPM: sinus rhythm pacing medium, AFPM: atrial fibrillation pacing medium.

To evaluate whether cardiomyocyte specific overexpression of miR-21 induces fibrosis, the medium of paced (AF and SR) and control HL1 cardiomyocytes was transferred to NIH/3T3 fibroblast and incubated. A fibrosis profiling array obtained from the fibroblasts showed strong induction of 24 transcripts associated with fibrosis pathways and collagen expression (Figure 3A). Most of the upregulated candidates were related to the TGF-β pathway (TGF-β 1 and its receptors, Ccl11/eotaxin-1- a promoter of TGF-β receptor, Integrin-av- an activator of TGF-β, Endoglin- a glycoprotein of the TGF-β receptor, inhibin- a member of the TGF-β superfamily of proteins and smad2 and 3- downstream actors of the TGF-β signaling). Moreover, two TGF-β pathway inhibitors, SMAD family member 7 (smad7) and decorin, were not detectable. Likewise, the fibrotic markers collagen1a, matrix-metalloproteinase-2 and 9 (MMP2 and 9), tissue inhibitor of metalloproteinases-3 (Timp3), connective tissue growth factor and marker for epithelial mesenchymal transition in fibroblast (Snail1) were upregulated.

TGF-β is key mediator of tissue fibrosis and a central regulator of fibrosis response (13). However, TGF-β has a pleiotropic effect and targeting it as therapeutic marker is complex (14). As fibrosis is the result of activated fibroblast that has been transformed to myofibroblast, we decided to analyse the marker of activated fibroblasts. For this, we examined alpha-smooth muscle actin (α-SMA) and connective tissue growth factor (Ctgf). α-SMA is a biomarker for myofibroblast (15) and Ctgf is a central mediator of fibrosis produced by fibroblast that stimulates myofibroblast function and cardiac fibrosis (16).

**Figure 4:**
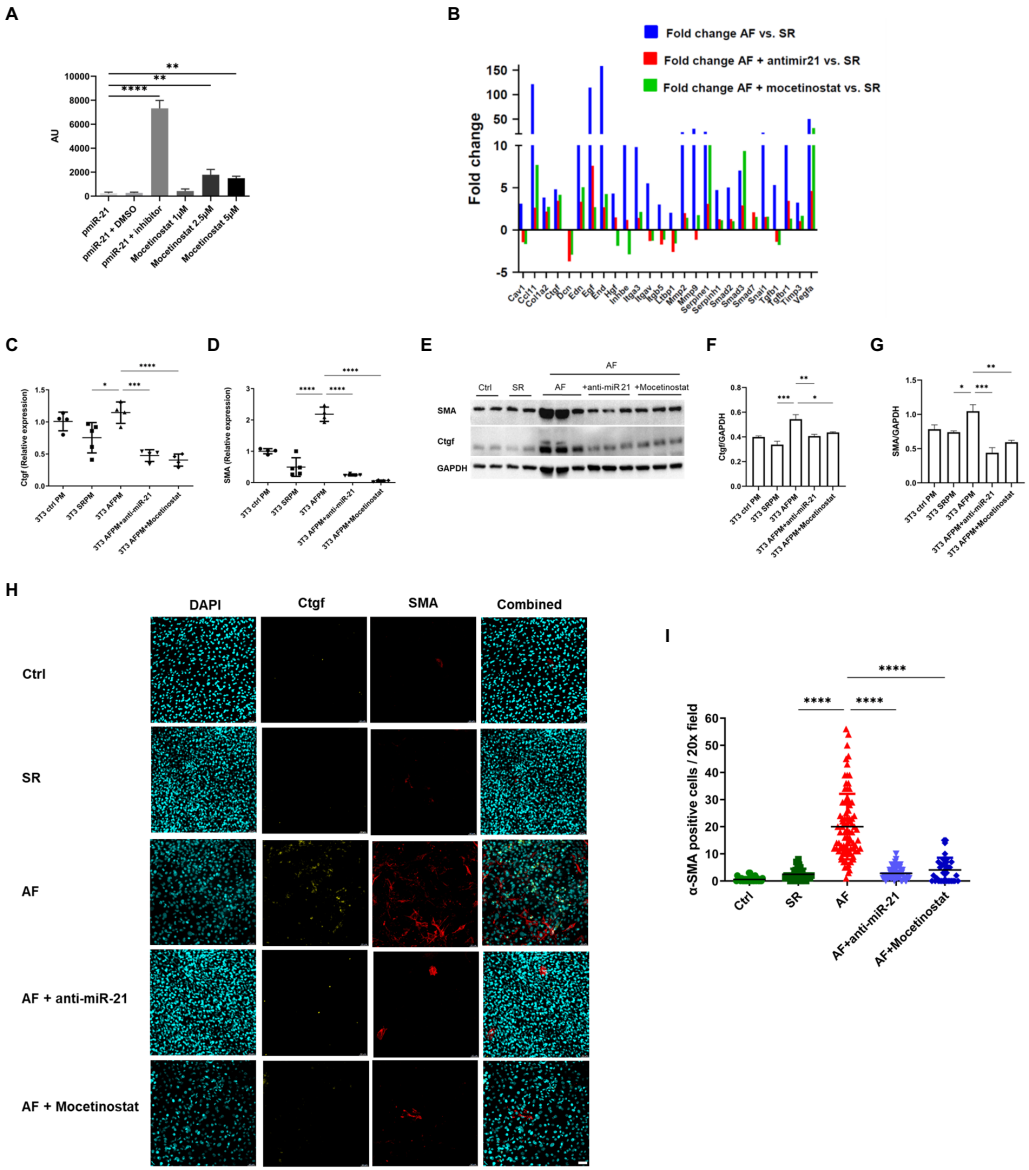
The HDAC1 inhibitor mocetinostat attenuates miR-21 expression and collagen production in AF. **(A)** A luciferase assay was performed with miR-21, miR-21 with DMSO, miR-21 and miR-21 inhibitor, different concentration of mocetinostat. **(B)** Mocetinostat attenuated activation of pro-fibrotic pathways and collagen expression in NIH/3T3 fibroblasts incubated with medium from AF- paced cells. Anti-mir-21, that specifically antagonizes miR-21 in the medium, showed similar effects. Each sample type represents a pool of one 6 well plate. Real time PCR **(C,D)**, Western blot analysis **(E–G)** and immunofluorescence staining **(H,I)** antibody against Ctgf and α-SMA and counterstaining with DAPI, representative pictures shown in **(H)** of the fibrosis markers Ctgf and SMA in fibroblasts incubated with SR and AF medium, could confirm the results of the profiler array. Scale bars = 50 μm. **(A,C–G)** Representative data out of three similar experiments. Data are expressed as mean ± SEM. ****p < 0.0001, ***p < 0.001, **p < 0.01, *p < 0.05. Abbreviations same as Figure 3.

Fibrosis profiler array data was confirmed by RT-PCR, western blot and immunofluorescence. mRNA expression of α-SMA and Ctgf was significantly increased in fibroblasts that received the AF paced medium compared to cells that received SR medium (4.5 fold for α-SMA, *p* < 0.0001 and 1.5 fold for Ctgf, *p* < 0.05; Figures 3B,C). The corresponding proteins were also upregulated, as shown by western blot and immunofluorescence (Figures 4E–I).

### 3.4. The HDAC1 inhibitor mocetinostat and anti-miR-21 attenuates miR-21 release in AF and fibrosis development

An *in-vitro* screen was performed with selected FDA- approved drugs using a miR-21 luciferase promoter assay (Supplementary Figure 2). Among the screened drugs, the HDAC1 inhibitor mocetinostat was found to significantly inhibit miR-21 expression (Figure 4A) in a dose dependent manner. Therefore, we decided to further evaluate the potential of mocetinostat to intervene the miR-21 pathway and curb the fibrotic pathway. Mocetinostat at a concentration of 1 μM reduced miR-21 activity by 1.82 fold, whereas a 2.5 μM concentration led to 7.64 fold and 5 μM concentration to a 6.4 fold reduction in miR-21 activity. PCR profiling array of fibroblasts incubated with AF, SR or control medium and later treated with either mocetinostat or anti-miR-21 showed that inhibition of miR-21 reversed the activation of the pro-fibrotic transcripts with the exception of Smad3 and restored the expression of the TGF-β pathway inhibitors smad7 and decorin (Figure 4B). Mocetinostat led to a reduction of Ctgf expression (2.8 fold, *p* < 0.001) and α-SMA expression (around 20 fold, *p* < 0.0001) in fibroblasts incubated with AF-paced medium (Figures 4C,D). The anti-fibrotic effects of mocetinostat and anti-miR-21 could also be confirmed by western blot and immunofluorescence (Figures 4E–I, Supplementary Figure 3). Mocetinostat reduced Ctgf by 20% (*p* < 0.05) and α-SMA expression by 43% (*p* < 0.01) in fibroblasts incubated with AF-paced medium (Figures 4F,G).

## 4. Discussion

Atrial fibrillation is a complex disease with multiple pathomechanisms. A generally recognized model of AF states that ectopic foci located in the pulmonary veins initiate the arrhythmia. AF then leads to adverse atrial remodeling with collagen production, creating micro-re-entries in the left atrium that sustain the disease. The theory that “AF begets AF” is a well-established clinical observation, however, exact mechanisms of LA fibrosis development in AF are poorly understood.

Our study shows that:

1. Circulating miR-21 correlates with left atrial fibrosis in a large cohort of AF patients undergoing catheter ablation
2. miR-21 also correlates with event-free survival after ablation and is a reliable predictor for treatment outcome
3. miR-21 is released *in-vitro* from cultured cardiomyocytes under tachyarrhythmic conditions
4. miR-21 stimulates fibroblasts in collagen expression and contributes to adverse atrial remodeling
5. Atrial remodeling can be mitigated *in-vitro* by the HDAC1-inhibitor mocetinostat, which might serve as a novel therapeutic option for AF patients

To investigate the mechanisms involved in miR-21 mediated atrial fibrosis, we utilized an established *in-vitro* AF model (17). We found increased expression and release of miR-21 to the culture medium after tachyarrhythmic pacing. This positively correlates with the increased expression of miR-21 in the patient arterial serum. Interestingly, transferring the medium of the paced cardiomyocytes to cultured fibroblasts resulted in activation of profibrotic pathways and collagen expression. Several studies confirm miR-21 mediated transformation of cardiac fibroblast to myofibroblast leading to cardiac fibrosis by targeting many pathways: Jagged1, cell adhesion molecule 1 (CADM1)/signal transducer and activator of transcription 3 (STAT3), transforming growth factor β (TGF-β1)/Smad7 and phosphatase and tensin homologue (PTEN) pathway (10, 18–20). miR-21 is also known to target TGF-β1 thereby increasing collagen I, smooth muscle actin and connective tissue growth factor in both mRNA and protein levels (10, 20).

**Figure 5:**
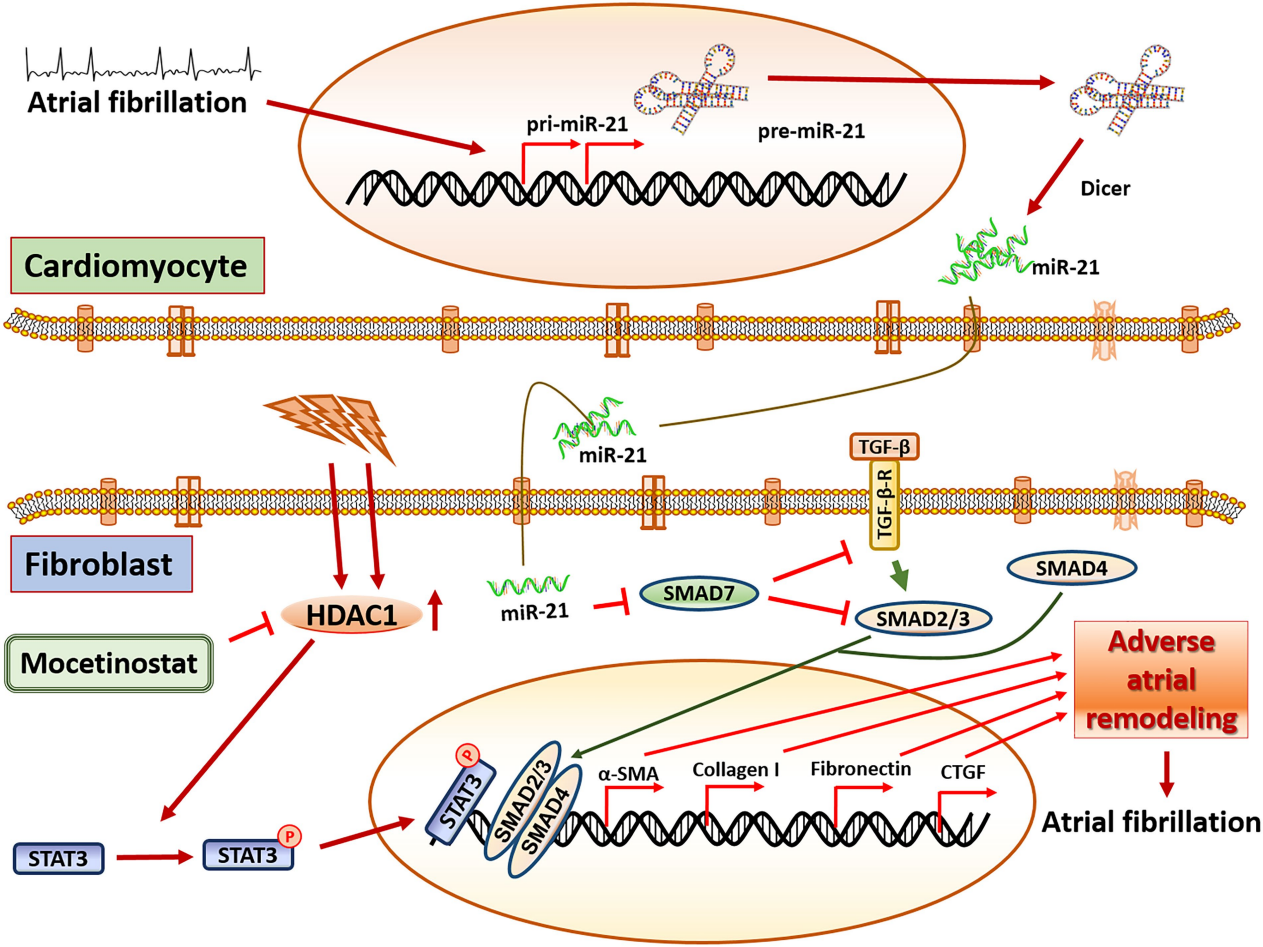
Central illustration. The proposed pathway in miR-21 mediated adverse atrial remodeling. miR-21 is released from cardiomyocytes under tachyarrhythmic conditions and triggers pro-fibrotic pathways in fibroblasts, mainly through the SMAD7/TGF-b pathway. Mocetinostat, an HDAC1 inhibitor, attenuates miR-21 dependent adverse atrial remodeling and collagen creation by antagonizing the STAT3-pathway. Tgf-β: transforming growth factor beta 1, Tgf-β-R: transforming growth factor beta receptor, HDAC1: Histone deacetylase 1, Smad7: SMAD family member 7, Smad2/3: SMAD family member 2/3, Smad4: SMAD family member 4, STAT3: signal transducer and activator of transcription 3, α-SMA: alpha smooth muscle actin, CTGF: connective tissue growth factor.

MiR-21 as a biomarker in AF is well established (21–23). For example, a study by McManus et al. as well as a study by Dawson et al. found lower miR-21 concentrations in patients with AF compared to healthy controls (21, 23). Interestingly, our previous study with blood taken directly from the left atrium (11) and our current study with blood from a peripheral artery (radial artery) showed a positive correlation between miR-21 concentration and left atrial fibrosis. However, in clinical routine, atrial or arterial blood collection seems barely feasible. Therefore, in addition to arterial blood, we also analysed miR-21 expression in peripheral venous blood in our patients. Interestingly, circulating miR-21 concentration in venous blood was different from arterial blood (Supplementary Figures 1A,B). Although we do not have a conclusive explanation for this discrepancy, we hypothesize that different miR-21 expression levels depend upon tissue typecasts or specific area from where the blood samples were collected. This supports the notion that arterial and venous blood have different miRNA characteristics (24, 25). Further, miR-21 is a hypoxia regulated element (26) and there could be differential expression of miRNA in response to oxygen concentration. For example, Xu et al. (24) have shown that miRNA expression profiles are not identical in arterial and venous plasma and arterial plasma miRNA had a higher correlation with the tissue miRNA expression profile. It is also important to mention that the study population was older, had more LA fibrosis, larger left atrias, higher CHA2DS2-VASc scores and a lower LV-EF compared to the AF population in our previous study (11), which might have influenced miR-21 expression. It would therefore be of interest to further explore the mechanism how miR-21 or other micro-RNAs are regulated in the body (Figure 5).

Pulmonary vein isolation, an interventional approach to eliminate triggers that induce AF, is the cornerstone in AF treatment with considerable success of long-term freedom from the arrhythmia (27, 28). However, with progression of the disease and increasing adverse atrial remodeling, durable restoration of SR gets increasingly difficult and long-term treatment outcomes decrease dramatically (7). Although there seems to be a linkage between increased atrial fibrosis and more advanced stages of AF, targeting areas with atrial fibrosis for substrate ablation have failed to show a clinical benefit with current ablation strategy (29, 30). It would therefore be of immense importance to establish a method to stop or reverse adverse atrial remodeling and substrate generation. However, such a treatment option does not exist so far.

In our study, we raise the hypothesis that mocetinostat could act as a potential modulator of atrial cardiomyopathy. The role of HDAC and its inhibition affecting the AF has been already shown (31–33). However, the mechanism through which mocetinostat acts in fibrosis has not been explored yet. Epigenetic modulation plays an important role in activated fibroblasts. Histone deacetylation which is associated with gene repression, is increased and treatment with HDAC inhibitors suppresses the proliferation and epithelial mesenchymal transition of the fibroblasts. Consequently, HDAC inhibitors can reduce extracellular matrix production and influence the inflammatory response (TGF-β pathway) (34, 35). Yoon et al. (36) showed that in murine cardiac fibroblasts, HDAC inhibitors attenuated myofibroblast differentiation thereby impeding the development of hypertrophy and cardiac fibrosis. In our study, we could confirm the anti-fibrotic effects of HDAC1 inhibitor mocetinostat in activated fibroblasts by inhibiting miR-21 expression. Consistent with our data, mocetinostat decreased the expression of α-SMA, collagen III and matrix-metalloproteinase-2 and was able to reverse cardiac fibrosis (37). Mocetinostat, which is currently under clinical investigation for the treatment of various cancers including follicular lymphoma, Hodgkin’s lymphoma and Leiomyosarcoma (38–40), might therefore also be a therapeutic option to stop or reverse disease progression in AF.

We acknowledge following limitations in our study: Different strategies have been used for AF ablation (PVI alone, +/− roof line, mitral isthmus line, CFAE ablation, CTI line), which might have influenced treatment outcome. The ablation strategy was determined by the operator (without randomisation) and mainly depended on the presence of LVAs and inducibility of regular tachycardias. Our study was therefore not designed to compare different ablation strategies and treatment outcome has not been differentiated between the ablation techniques.

We have not assessed other markers of atrial cardiomyopathy that are currently under investigation (e.g., fibroblast growth factor 23 and N-terminal-pro hormone B-type natriuretic peptide). Likewise, delayed enhancement magnetic resonance imaging (DE-MRI) of the left atrium to visualize left atrial fibrosis was not performed. For our *in-vitro* studies, cardiomyocytes and fibroblasts were cultured separately which may not represent an exact *in-vivo* scenario. Direct co-culturing would have been preferable, but was not feasible, since fibroblasts have a higher proliferation rate and outgrew the HL1 cardiomyocytes, which made it complicated to execute our protocol of pacing and incubating the cells for 72 h. Furthermore, production of miR-21 *in-vivo* might have a different source than cardiomyocytes and its effects might also be beyond fibroblasts. Finally, we focused on the effect of mocetinostat on fibrosis induced by HL-1 derived miR-21. We have not included a miRNA mimic to determine the post-transcriptional regulatory relationship between miR-21 and fibrosis.

In conclusion, our results confirm that miR-21 plays an important role as a biomarker for atrial fibrosis and for prediction of treatment outcome after AF catheter ablation. We also found that miR-21 is involved in the pathogenesis of disease progression and substrate creation by mediating a cross-talk between cardiomyocytes and fibroblasts. We suggest mocetinostat as a potential treatment option that might attenuate atrial fibrosis development, interfering with the vicious circle between arrhythmia and adverse atrial remodeling.

## Data availability statement

The original contributions presented in the study are included in the article/Supplementary material, further inquiries can be directed to the corresponding author.

## Ethics statement

The studies involving human participants were reviewed and approved by Ethics committee of the medical faculty of RWTH Aachen. The patients/participants provided their written informed consent to participate in this study.

## Author contributions

KP and MG contributed to the study design, data analysis and interpretation and manuscript preparation. KP, PN, BN, and CM contributed to the experimental works, data analysis, and interpretation. AS-Y, ME, MZ, AN, and NM contributed with significant intellectual work and manuscript preparation. All authors contributed to the article and approved the submitted version.

## Funding

This work was supported by the German Research Foundation (DFG) grant (GR 3411/5-1) to MG and Bridging fund (Eberhard Karls University of Tübingen) to KP.

## Conflict of interest

The authors declare that the research was conducted in the absence of any commercial or financial relationships that could be construed as a potential conflict of interest.

## Publisher’s note

All claims expressed in this article are solely those of the authors and do not necessarily represent those of their affiliated organizations, or those of the publisher, the editors and the reviewers. Any product that may be evaluated in this article, or claim that may be made by its manufacturer, is not guaranteed or endorsed by the publisher.
