# Supplementary material for: MicroRNA-21 mediated cross-talk between cardiomyocytes and fibroblasts in patients with atrial fibrillation
Source: Front Cardiovasc Med. 2023 Feb 17;10:1056134. doi: 10.3389/fcvm.2023.1056134 (PMC9982105; doi:10.3389/fcvm.2023.1056134)
Supplement: Supplementary file 1 [file Data_Sheet_1.pdf]

Supplementary Material

1 Supplementary Data

Expanded Materials and Methods

Follow-up: In all patients, serial 7-day ECGs were performed 3, 6, and 12 months after the procedure. In addition, patients were instructed to seek ECG documentation when symptoms suggestive of AF occurred. Recurrences were defined as documented AF or atrial tachycardia (both symptomatic and asymptomatic, documented in a 7-day Holter ECG with a duration >30 s or in a 12-lead ECG after a blanking period of 3 months).

Cell culture: The HL1 mouse atrial cardiomyocyte were cultured using Claycomb medium supplemented with 10% fetal bovine serum, 2mM L-Glutamine, 0.1mM norepinephrine and 1% penicillin/streptomycin (100 units/ml penicillin and 100µg/ml streptomycin) and cultured as described in the protocol (1).

The NIH/3T3 mouse embryonic fibroblast were cultured in dulbecco's modified eagle's medium (4mM L-glutamine, 4500mg/l glucose, 1500mg/l sodium bicarbonate and 1mM sodium pyruvate),10% bovine calf serum and 1% penicillin/streptomycin.

The HeLa cells were cultured in dulbecco's modified eagle's medium (4mM L-glutamine, 4500mg/l glucose, 1500mg/l sodium bicarbonate and 1mM sodium pyruvate),10% fetal bovine serum and 1% penicillin/streptomycin.

Cloning of the miR-21 binding site in the pmirGlo luciferase vector: A sequence including the binding site of miR-21 was annealed to its complementary sequence and the ds DNA was ligated in the 3'UTR of firefly luciferase in pmirGlo vector (Promega) digested with XbaI and MssI. The ligation products were transformed in One Shot TOP10 chemically competent E. coli (Thermofisher) and positive colonies were identified over digestion of the plasmids with NotI. pmiR21GLO plasmid was purified with NucleoBond kit (NucleoBond® PC 500 EF, Endotoxin Free plasmid DNA purification, Machenery-Nagel).

Loss of function/Gain of function of miR-21-5p and drug treatment: The HeLa cells were transfected in 96-well plates with pmir21GLO with lipofectamine 2000 (Thermofisher). In addition to this vector, transfection was done either with anti-miR-21 at concentration 50µM or with different concentration of FDA approved drugs: entresto, mocetinostat, valsartan, sotalex, MPTOE14, spironolactone, and eplerenone. Additionally, Hela cells were transfected with LightSwitch™ miRNA-21-5p target Reporter (Active Motif, S880173) and LightSwitch™ GAPDH 3'UTR control (positive control) (Active Motif, S801378), LightSwitch™ Empty 3'UTR control (positive transfection control and negative control for miRNA signalling) (Active Motif, S890005), LightSwitch™ miRNA mimic has-miR-21-5p (Active Motif, MIM0052), LightSwitch™ miRNA mimic non-targeting_2 (as a control for mimic) (Active Motif, MIM9002), miR-21- 5p inhibitor (Qiagen) and miR-21 inhibitor NC5 negative control (Qiagen)

NIH/3T3 cells were transfected with Lipofectamine® RNAiMAX reagent (Invitrogen) and miR-21-5p inhibitor (Qiagen) (50µM) or miR-21 inhibitor NC5 negative control (25nM) according to the manufacturer's protocol. Mocetinostat (2.5µM) diluted in the medium from paced HL1 cells was added to the NIH/3T3 cells. Cells were harvested after 72 hours for gene and protein expression analysis.

<u>Luciferase assay:</u> The expression of firefly luciferase assay was measured using either Dual-Luciferase® reporter assay system (Promega, E1910) or LightSwitch™ Luciferase Assay kit (Active Motif, 32032) following the kits protocol. Reading was done in microplate reader Infinite M200 (Tecan).

<u>RNA isolation and quantitative real- time PCR:</u> RNA isolation from the patient serum, cell pellet and medium (cultured cells) were done by using miRNeasy mini kit (Qiagen 217004). For patient serum and medium: 200□l of serum/medium was mixed with 1400□l of Qiazol. For cell pellet: homogenisation was done using QIA shredder (Qiagen 79656). All other steps were according to the protocol. cDNA isolation for microRNA was done by using miRCURY LNA RT kit (Qiagen 339340). Cel-miR-39 was used as external spike-in reference (Qiagen 339306). hsa-miR-23a-3p (Qiagen 339306) and hsa-miR-23b-3p (Qiagen 339306) were selected as reference gene and their geometric mean was used for the qPCR analysis. For the analysis of fibrotic genes, cells were treated with DNase by using Rnase free DNase set (Qiagen 79254) to avoid the contamination of genomic DNA. cDNA preparation from RNA, isolated from NIH/3T3 fibroblasts, was done using iscript cDNA synthesis kit (Bio-rad 1708891). viiA7 real-time PCR System (Applied Biosystem) was used to conduct real time PCR experiments.

Primers used:

Cel-miR-39: cel-miR-39-3p miRCURY LNA miRNA PCR Assay (YP00203952/ 339306)

has-miR-21-5p: has-miR-21-5p miRCURY LNA miRNA PCR Assay (YP00204230/339306)

hsa-miR-23a-3p: hsa-miR-23a-3p miRCURY LNA miRNA PCR Assay (YP00204772/339306)

hsa-miR-23b-3p: hsa-miR-23b-3p miRCURY LNA miRNA PCR Assay (YP00204790/339306)

Mouse Ctgf forward: GGAGGAAAACATTAAGAAGGGC

Mouse Ctgf reverse: AAGCTCAAACTTGACAGGCT

Mouse α-SMA forward: CTCCCTGGAGAAGAGCTACG

Mouse α-SMA reverse: AAATGTGACACCATCCCAATGA

Mouse TBP forward: CAGTGCCCAGCATCACTATT

Mouse TBP reverse: GCATAAGGTGGAAGGCTGTT

Expression profiling: 0.5µg RNA isolated from the fibroblasts out of the pacing experiments (one 6-well-plate was pooled) and fulfilling the requisites of purity, was used as template for a profiler PCR array (RT2 profiler PCR Array mouse fibrosis 96 well format, Qiagen). Expression profiling was performed according to the manufacturer's guideline.

Protein isolation and western blot: The treated NIH/3T3 cells with medium of controlled or paced HL-1 cells were washed twice with Ca2+-free PBS and lysed with Pierce RIPA buffer (Thermofisher, 89900) containing PhosSTOPTM (Sigma Aldrich, 4906837001) and cOmpleteTM Protease Inhibitor cocktail (Sigma Aldrich, 11697498001). The mixture was sonicated and centrifuged at 14800rpm for 10 minutes at 4°C. The supernatant was collected and protein concentration determined by bicinchoninic acid (BCA) assay. 10µg of protein was mixed with 4x sample loading dye, denatured at 95°C for 6 minutes and separated by sodium dodecyl sulphate-polyacrylamide gel electrophoresis (SDS-PAGE) using Bio-Rad western blot equipments. The conditions were adjusted at 80 volts through 5% stacking gel followed by 100 volts through 10% resolving gel for approximately 2 hours. Separated proteins were blotted on nitrocellulose membrane and blocked with 5% milk powder in 1x TBST buffer at room temperature for 60 minutes. The primary antibodies used were: CTGF (1:500) (Proteintech, 23936-1-AP), Dako monoclonal mouse anti-human α-SMA antibody clone 1A4 (1:1000) (M0851) and GAPDH (1:20000) (Proteintech, 10494-1-AP). The secondary antibodies were anti-rabbit IgG HRP-linked antibody (1:10000) (Cell signaling, 7074S) and anti-mouse IgG HRP-linked antibody (1:10000) (Cell signalling, 7076S). The antibodies were prepared in 2% bovine serum albumin (BSA) and incubated at room temperature for 60 minutes each. The blots were washed with TBST buffer between each incubation steps. Protein expressions were detected using SuperSignalTM West Pico PLUS chemiluminescent substrate (Thermofisher Scientific) in Bio-Rad western blot imager.

Immunocytochemistry: Cells were seeded in 12 well plate with sterile coverslips. Unless stated all the steps were performed at room temperature. Cells were fixed in 4% formaldehyde for 20 minutes. Cells were permeabilized with 0.1% Triton X-100 in PBS for 10 minutes. Blocking was done in 5% BSA diluted with PBS for 30 minutes. Overnight incubation at 4°C was done with primary antibodies against alpha smooth muscle actin Dako monoclonal mouse anti-human α-SMA antibody clone 1A4 (M0851) (1:100) followed by washing and incubation with the secondary antibody CyTM3 Affinipure donkey anti-Ms IgG (Jackson Immunoresources, 715-165-150) (1:300). After blocking again with 5% BSA diluted with PBS for 30 minutes, second primary antibody against connective tissue growth factor (Proteintech, 23936-1-AP) (1:50) was used and incubated overnight at 4°C. After washing the cells with PBS, incubation with the secondary antibody FITC Affinipure donkey anti-rabbit IgG (Jackson Immunoresearch, 711-095-152) (1:100) was performed. Nuclear counterstain was done using 4'6-diamidino-2-phenylindole (DAPI) (Invitrogen, D1306). The coverslip was taken out and fixed in the slide using a drop of fluorescence mounting medium. Cells were imaged using fluorescence microscope Leica Dmi8.

2 Supplementary Figures

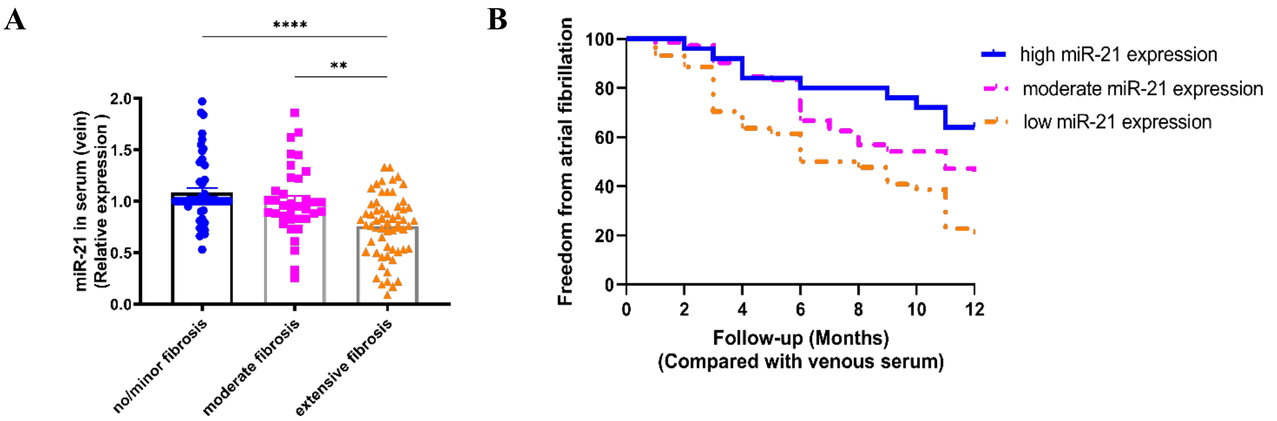

Supplementary Figure 1: Correlation of miR-21 in venous serum and LVAs. (A) Circulating miR-21 in venous serum correlates negatively with left atrial LVAs. Patients with a low miR-21 serum concentration had significantly more LVAs; patients with a very high miR-21 serum concentration had no or only mild left atrial fibrosis. n=36-58. **(B)** Circulating miR-21 in venous serum correlates with treatment outcome after catheter ablation. 64% of patients with high miR-21 serum levels were in stable SR 12 months after ablation, whereas 45.8% with moderate miR-21 concentration and 20.5% with low miR-21 concentration were free from AF 12 months after ablation. n=25-72. Data are expressed as mean±SEM. ****$p<0.0001$, **$p<0.01$. LVAs = low voltage area

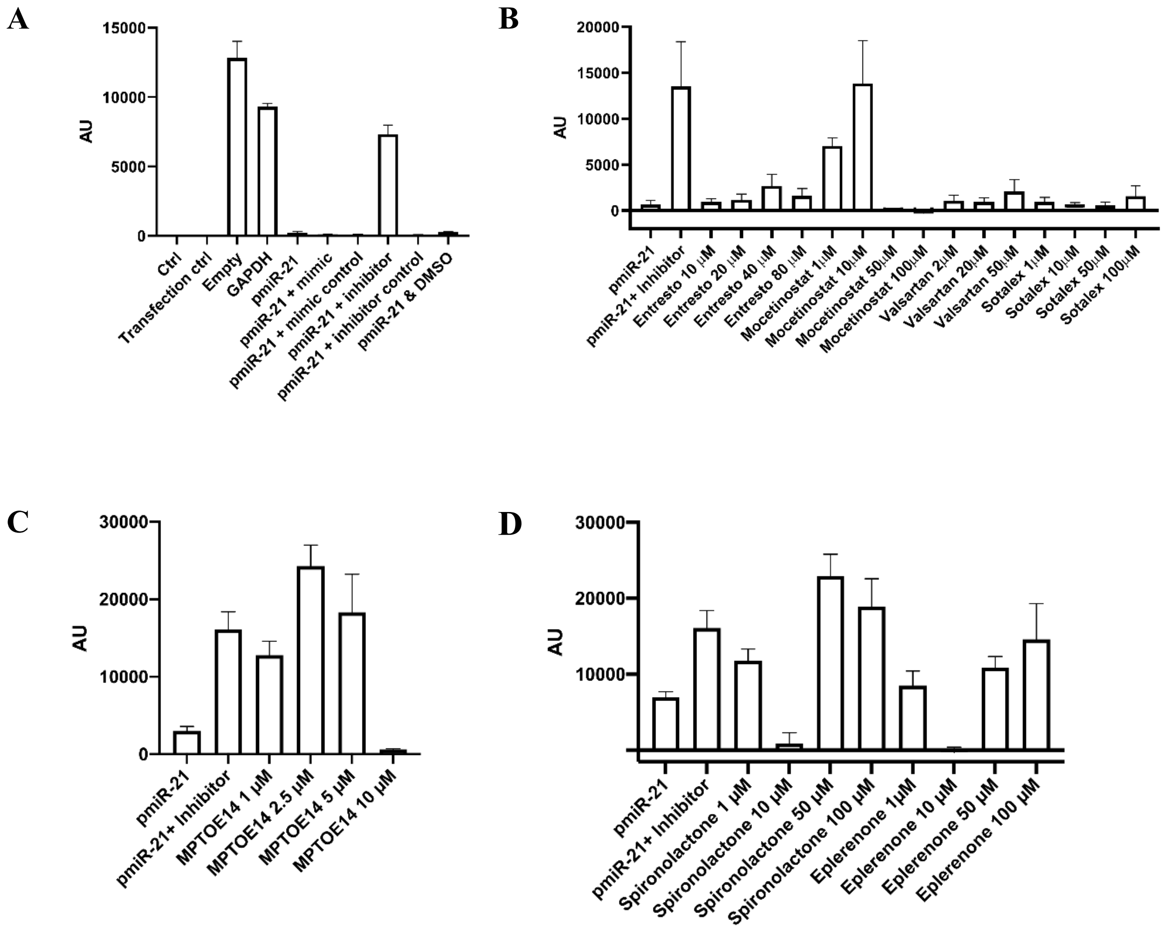

Supplementary Figure 2. miR-21 specificity and drug screening to select the drug which inhibits miR-21 expression. (A) Luciferase assay was performed in Hela cells 48 hours after transfection with LightSwitch vectors, mimics and inhibitors to look for the specificity of miR-21 inhibitor. Ctrl: no transfection, Transfection ctrl: transfection reagent only, Empty: positive transfection control and negative control for miRNA signaling, GAPDH: positive control, pmiR-21: miR-21 target reporter vector, mimic: mimic of miR-21, mimic control: control used for mimic, inhibitor: miR-21 inhibitor, inhibitor control: control for inhibitor, DMSO: Dimethyl sulfoxide. Transfection done with LightSwitch kits. **(B-D)** Luciferase assay (Promega) was performed in Hela cells 48 hour after transfection with plasmid containing miR-21. Following drugs were screened:

entresto, mocetinostat, vvalsartan, sotalex, MPTOE14, spironolactone, eplerenone. **(A-D)**. Representative data out of 3 similar experiments. Data are expressed as mean±SEM.

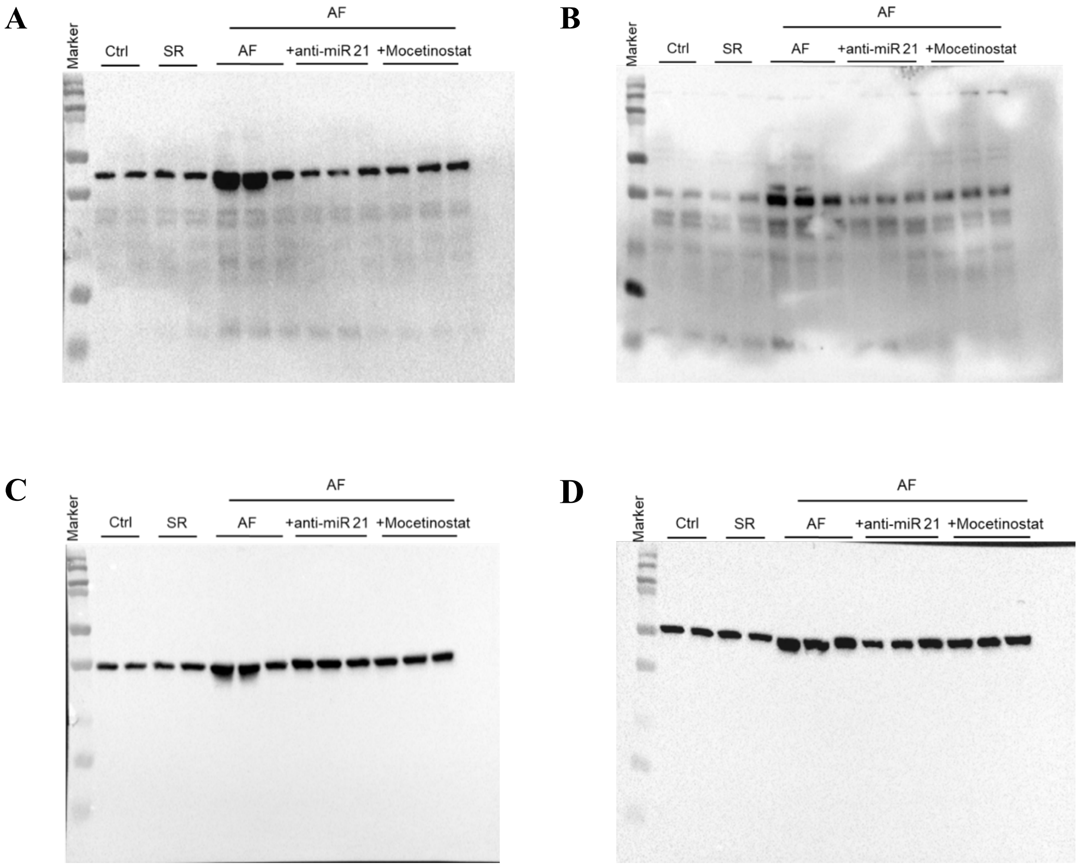

E

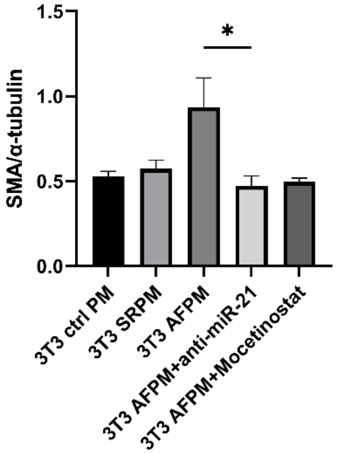

F

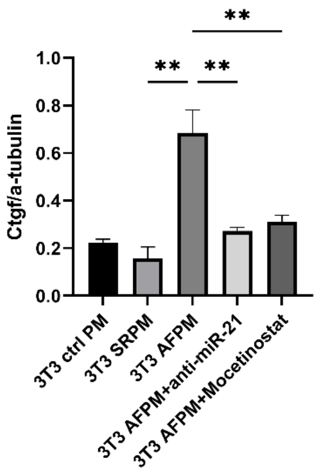

Supplementary Figure 3. Raw and uncropped western blot gels scans. (A) Smooth muscle actin (SMA). (B) Connective tissue growth factor (Ctgf). (C) Glyceraldehyde 3-phosphate dehydrogenase (GAPDH). (D) alpha-tubulin (As a second reference gene). (E-F) Densitometry analysis of SMA and Ctgf compared to α-tubulin. Data are expressed as mean±SEM. **p<0.01, *p<0.05.

Reference

1. Claycomb WC, Lanson NA Jr, Stallworth BS, Egeland DB, Delcarpio JB, Bahinski A, et al. HL-1 cells : A cardiac muscle cell line that contracts and retains phenotypic characteristics of the adult cardiomyocyte. *Proc Natl Acad Sci U S A*. (1998) 95:2979–2984. doi: 10.1073/pnas.95.6.2979.
